# β-TrCP-mediated ubiquitination and degradation of liver-enriched transcription factor CREB-H

**DOI:** 10.1038/srep23938

**Published:** 2016-03-31

**Authors:** Yun Cheng, Wei-Wei Gao, Hei-Man Vincent Tang, Jian-Jun Deng, Chi-Ming Wong, Chi-Ping Chan, Dong-Yan Jin

**Affiliations:** 1 School of Biomedical Sciences, The University of Hong Kong, Pokfulam, Hong Kong; 2 State Key Laboratory for Liver Research, The University of Hong Kong, Pokfulam, Hong Kong; 3 Shenzhen Institute of Research and Innovation, The University of Hong Kong, Shenzhen, China; 4 Department of Food Science and Engineering, College of Chemical Engineering, Northwestern University, Xi’an 710069, China; 5 Department of Medicine and State Key Laboratory of Pharmaceutical Biotechnology, The University of Hong Kong, Pokfulam, Hong Kong

## Abstract

CREB-H is an endoplasmic reticulum-resident bZIP transcription factor which critically regulates lipid homeostasis and gluconeogenesis in the liver. CREB-H is proteolytically activated by regulated intramembrane proteolysis to generate a C-terminally truncated form known as CREB-H-ΔTC, which translocates to the nucleus to activate target gene expression. CREB-H-ΔTC is a fast turnover protein but the mechanism governing its destruction was not well understood. In this study, we report on β-TrCP-dependent ubiquitination and proteasomal degradation of CREB-H-ΔTC. The degradation of CREB-H-ΔTC was mediated by lysine 48-linked polyubiquitination and could be inhibited by proteasome inhibitor. CREB-H-ΔTC physically interacted with β-TrCP, a substrate recognition subunit of the SCF^β-TrCP^ E3 ubiquitin ligase. Forced expression of β-TrCP increased the polyubiquitination and decreased the stability of CREB-H-ΔTC, whereas knockdown of β-TrCP had the opposite effect. An evolutionarily conserved sequence, SDSGIS, was identified in CREB-H-ΔTC, which functioned as the β-TrCP-binding motif. CREB-H-ΔTC lacking this motif was stabilized and resistant to β-TrCP-induced polyubiquitination. This motif was a phosphodegron and its phosphorylation was required for β-TrCP recognition. Furthermore, two inhibitory phosphorylation sites close to the phosphodegron were identified. Taken together, our work revealed a new intracellular signaling pathway that controls ubiquitination and degradation of the active form of CREB-H transcription factor.

CREB-H, also known as cAMP responsive element-binding protein 3-like 3 (CREB3L3), is an endoplasmic reticulum (ER) membrane-bound transcription factor which is mainly expressed in the liver and small intestine^1^,^2^. CREB-H belongs to the CREB3 subfamily of bZIP proteins and the other subfamily members are CREB3/Luman, CREB3L1/OASIS, CREB3L2/BBF2H7 and CREB3L4/AIBZIP^3^. CREB-H is activated by regulated intramembrane proteolysis to liberate a constitutively active form known as CREB-H-ΔTC, which contains the N-terminal transactivation and DNA-binding domains but lacks the C-terminal transmembrane and luminal domains. CREB-H-ΔTC translocates into the nucleus to activate target gene expression^4^,^5^. Currently, it has been shown that CREB-H mRNA synthesis is induced by metabolic stimuli, such as fatty acids, fasting, peroxisome proliferator-activated receptor α (PPAR-α) and glucocorticoids^6^,^7^,^8^. CREB-H and other transcription factors of the CREB3 subfamily are structurally related to ATF6 and are therefore also thought to be activated proteolytically in response to ER stress^9^. However, this remains controversial because the generation of CREB-H-ΔTC is not activated by ER stressors such as thapsigargin and tunicamycin^10^. On the contrary, tunicamycin perturbs N-linked glycosylation in the luminal domain of CREB-H, which is required for optimal proteolytic activation^11^.

CREB-H is a multifunctional protein which regulates lipid metabolism, iron homeostasis, gluconeogenesis, cell secretion, cell growth and acute phase response^4^,^5^,^7^,^12^,^13^,^14^. Growing evidence suggests that CREB-H plays important roles in lipid metabolism and whole-body energy homeostasis^15^,^16^. Transcriptomic analysis has revealed a subset of CREB-H-regulated genes that are critically involved in lipid metabolic processes^12^,^17^,^18^. The CREB-H knockout mice exhibit marked hypertriglyceridemia (HTG), which is caused by impaired lipoprotein lipase-mediated triglyceride clearance^12^. Heterozygous non-synonymous or insertional mutations of CREB-H have also been found in HTG patients^12^,^19^. On the other hand, expression of CREB-H-ΔTC in mouse models of obesity and diabetes has been shown to have beneficial effects, which are mediated in part through the activation of fibroblast growth factor 21 (FGF21), a hepatokine and a potential therapeutic agent for obesity-related medical conditions^16^,^18^. CREB-H also promotes lipid droplet formation and triglyceride storage in liver tissues by increasing the expression of a lipid droplet-associated protein named fat-specific protein 27 β (FSP27β)^20^. In addition, adipose tissue-derived fatty acids are known to regulate the expression of FGF21 and other genes through CREB-H^21^. Other CREB-H-regulated genes include APOA4, which encodes apolipoprotein A4 involved in the activation of lipoprotein lipase^10^,^12^, and MMP13, which encodes matrix metallopeptidase 13 controlling extracellular matrix remodeling, cell secretion and mineral homeostasis^14^. CREB-H has a significant impact on hepatic function and its activity has to be tightly regulated. CREB-H-ΔTC is the active form of CREB-H that activates target genes directly. We and others have previously found that CREB-H-ΔTC is a short-lived protein^4^,^5^,^11^,^22^. This might help to ensure that the activation of its target genes is transient and the impact on lipid homeostasis is not long-lasting. However, exactly how the degradation of CREB-H-ΔTC is regulated remains to be elucidated.

The ubiquitin-proteasome system is one major pathway for intracellular protein degradation^23^. A protein is ubiquitinated by three sequential enzymatic reactions catalyzed by ubiquitin-activating enzyme E1, ubiquitin-conjugating enzyme E2 and ubiquitin ligase E3. Whereas lysine 48-linked polyubiquitination normally leads to proteasome-mediated degradation of the target protein, lysine 63-linked polyubiquitination commonly results in alteration of protein function or subcellular localization^24^,^25^. In the ubiquitin-proteasome system, E3 ubiquitin ligase is the crucial component governing substrate specificity. More than 600 genes in the human genome encode putative E3 ubiquitin ligases^26^. The SKP1-CUL1-F-box (SCF) protein complexes are the largest family of E3 enzymes. Essential components of these complexes include scaffold protein cullin1 (CUL1), adaptor protein SKP1, RING finger protein RBX1 and variable F-box proteins. The F-box protein can recruit the specific substrate through its WD40 or LRR domain and bind with other SCF components through its F-box domain^27^,^28^. β-transducin repeat containing protein (β-TrCP) is a well-studied F-box protein with two paralogs in mammals, β-TrCP1 (Fbxw1) and β-TrCP2 (Fbxw11). The biological properties of the two paralogs are indistinguishable. Most β-TrCP substrates contain a consensus DSGϕxS degron, in which ϕ represents a hydrophobic amino acid and x could be any amino acid. The serine residues in the degron are phosphorylated. Recognition of the phosphodegron by β-TrCP leads to ubiquitination of the substrate^29^,^30^,^31^. β-TrCP regulates various cell signaling pathways by degrading key signal transducers such as β-catenin for Wnt signaling and IκBα for NF-κB signaling^32^,^33^. In addition, human papillomavirus type 16 and a few other viruses are capable of inhibiting β-TrCP function to facilitate their own replication in host cells^34^. On the other hand, human immunodeficiency virus type 1 Vpu protein can recruit β-TrCP to target its cellular receptor CD4 for degradation^35^.

Degradation of membrane-bound CREB-H is thought to be mediated through the ER-associated protein degradation (ERAD) pathway^36^. However, CREB-H-ΔTC is a nuclear protein with a fast turnover rate and its degradation is plausibly governed by other mechanisms. It is particularly noteworthy that CREB3L1 and CREB3L2 of the same subfamily are targeted by Fbxw7 ubiquitin ligase to undergo SCF-dependent degradation^37^. Both wild-type and active forms of CREB3L1 and CREB3L2 are degraded by Fbxw7. We therefore set out to investigate whether CREB-H-ΔTC might also be degraded by SCF. We found that CREB-H-ΔTC is indeed targeted by SCF^β-TrCP^. We also identified two degron-proximal inhibitory phosphorylation sites in CREB-H-ΔTC. When our study was completed, an independent group also reported SCF^β-TrCP^-mediated degradation of CREB-H-ΔTC^38^. The two studies corroborate with each other to demonstrate a new mechanism for regulated destruction of the active form of CREB-H.

## Results

### Degradation of CREB-H-ΔTC by the ubiquitin-proteasome pathway

**Figure 1:**
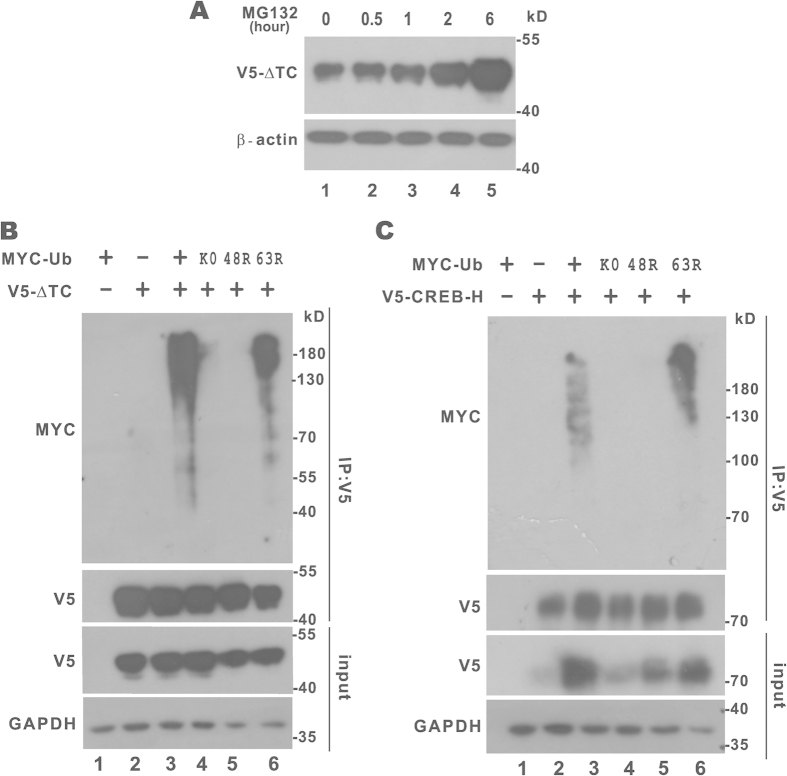
Degradation of CREB-H-ΔTC by the ubiquitin-proteasome pathway. (**A**) HEK293T cells expressing V5-tagged CREB-H-ΔTC (V5-ΔTC) were treated with 10 μM MG132 for 0.5, 1, 2 and 6 hours before harvest. Protein samples were separated by SDS-PAGE and probed with anti-V5 and anti-β-actin. (**B,C**) MYC-ubiquitin (MYC-Ub) and the indicated mutants were individually cotransfected with CREB-H-ΔTC or CREB-H into HEK293T cells. Immunoprecipitation was performed with anti-V5. Precipitates and inputs (10%) were analyzed by immunoblotting with anti-MYC. GAPDH was detected as an internal control. Ub: ubiquitin. K0: lysine free ubiquitin. 48R: ubiquitin with replacement of lysine 48 by arginine. 63R: ubiquitin with replacement of lysine 63 by arginine.

CREB-H is known to be stabilized by proteasome inhibition and degraded through the ERAD pathway^36^. However, membrane-bound CREB-H is transcriptionally inactive and CREB-H-ΔTC is the physiologically active form that activates transcription in the nucleus. We and others have found that CREB-H-ΔTC has a much shorter half-life than full-length CREB-H^4^,^5^,^22^. Because the active forms of CREB3L1 and CREB3L2 are also degraded through the ubiquitin-proteasome pathway^37^, we asked whether the fast turnover of CREB-H-ΔTC might also be governed by the same mechanism. Treatment of cells with proteasome inhibitor MG132 over a time course led to progressively increasing accumulation of CREB-H-ΔTC, indicating the requirement of the 26 S proteasome for CREB-H-ΔTC degradation (Fig. 1A, lanes 1–5).

To verify the polyubiquitination status of CREB-H-ΔTC and the type of ubiquitin linkage, we compared the ubiquitination patterns of CREB-H-ΔTC immunoprecipitated from HEK293T cells overexpressing wild-type and mutant ubiquitin. The ubiquitination smear of CREB-H-ΔTC was evident when wild-type ubiquitin but not its lysine-free mutant K0 was overexpressed (Fig. 1B, lanes 3 and 4). Furthermore, whereas the ubiquitination ladder of CREB-H-ΔTC was unaffected by ubiquitin mutant 63R, in which lysine 63 had been replaced by arginine, the 48R mutant of ubiquitin lacking lysine 48 did not support polyubiquitination of CREB-H-ΔTC (Fig. 1B, lanes 5 and 6). Thus, the polyubiquitin chain of CREB-H-ΔTC was lysine 48-linked. In addition, similar results in support of lysine 48-linked polyubiquitination were also obtained for full-length CREB-H (Fig. 1C). These data suggested that both CREB-H-ΔTC and CREB-H might be degraded through the ubiquitin-proteasome pathway.

### Identification of a putative β-TrCP recognizing motif in CREB-H-ΔTC

**Figure 2:**
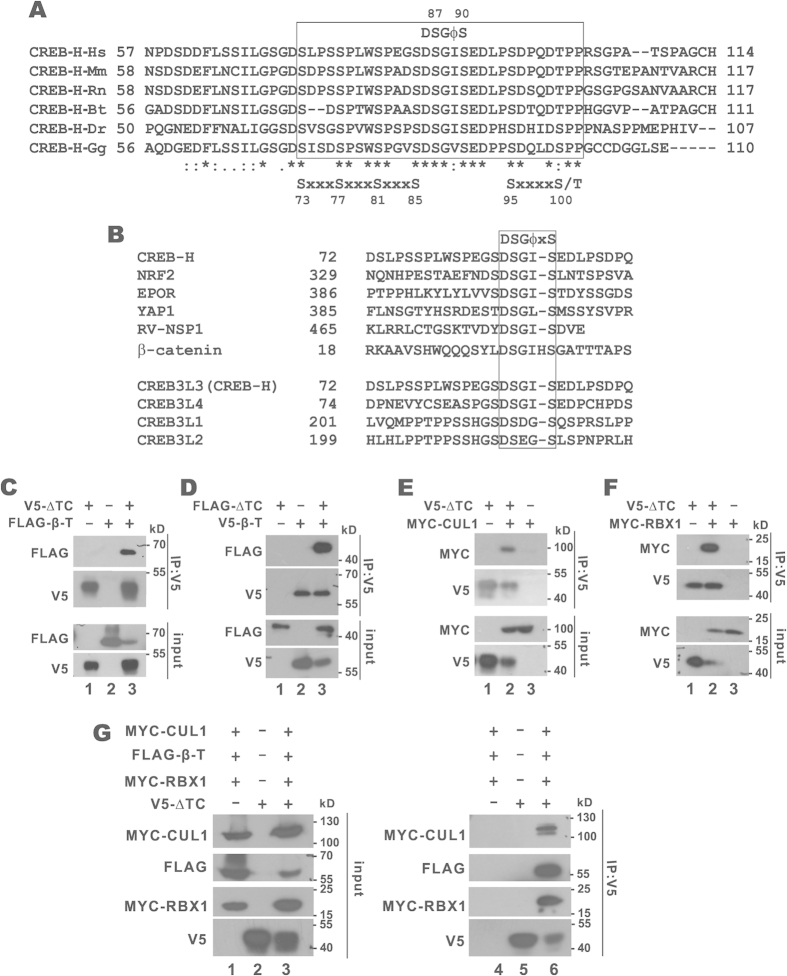
Interaction between CREB-H-ΔTC and SCF^β-TrCP^. (**A**) Sequence alignment of CREB-H homologs from different species. The predicted β-TrCP-recognizing motif (DSGϕS) and the adjacent highly conserved serine-threonine residues are framed and highlighted. Identical residues are denoted by asterisks (*), whereas colons (:) and periods (.) indicate strong and weak similarity, respectively. ϕ: hydrophobic residue. x: any residue. Hs: *Homo sapiens* (human). Mm: *Mus musculus* (house mouse). Rn: *Rattus norvegicus* (Norway rat). Bt: *Bos taurus* (bovine). Dr: *Danio rerio* (zebrafish). Gg: *Gallus gallus* (chicken). (**B**) Sequence alignment of CREB-H with known β-TrCP substrates (upper) and other CREB3 subfamily proteins (lower). The β-TrCP recognizing motif is indicated. EPOR: erythropoietin receptor. RV-NSP1: rotavirus nonstructural protein NSP1. (**C,D**) Interaction between CREB-H-ΔTC and β-TrCP. Reciprocal co-immunoprecipitation was performed with V5-CREB-H-ΔTC (V5-ΔTC) and FLAG-β-TrCP (FLAG-β-T) or FLAG-CREB-H-ΔTC (FLAG-ΔTC) and V5-β-TrCP (V5-β-T) expressed in HEK293T cells. Cell lysates were immunoprecipitated with anti-V5 and the precipitates were immunoblotted with anti-FLAG and anti-V5. The input cell lysates (10%) were also probed with anti-FLAG and anti-V5. (**E,F**) Interaction of CREB-H-ΔTC with CUL1 and RBX1. Lysates from transfected HEK293T cells were immunoprecipitated with anti-V5 and the precipitates were analyzed by immunoblotting with anti-V5 and anti-MYC. The input lysates (10%) were also detected with anti-V5 and anti-MYC. (**G**) Interaction of CREB-HΔTC with β-TrCP, CUL1 and RBX1. The anti-V5 immunoprecipitates were probed with anti-MYC, anti-FLAG and anti-V5.

Because CREB3L1 and CREB3L2 are degraded by SCF^37^, we sought to shed light on the identity of the E3 ubiquitin ligase for CREB-H-ΔTC by searching its sequence for recognition motifs by F-box proteins. Our bioinformatic analysis was fruitful. F-box protein β-TrCP recognizes a conserved degradation motif DSGϕxS with phosphoserines. Inspection of the CREB-H-ΔTC amino acid sequence revealed an evolutionarily conserved sequence, DSGIS, which resembles the canonical β-TrCP degradation motif (Fig. 2A). This putative β-TrCP recognizing motif located at residues 86–90 of CREB-H is identical or very similar to other β-TrCP degrons found in erythropoietin receptor, rotavirus nonstructural protein NSP1, transcription factor Nrf2, β-catenin and transcriptional co-activator YAP1 (Fig. 2B). Moreover, multiple alignment of CREB-H-ΔTC among different species revealed two additional clusters of evolutionarily conserved serine and threonine residues flanking the DSGIS motif: SxxxSxxxSxxxS located at residues 73–85 and SxxxxS/T located at residues 95–100 (Fig. 2A). Because phosphorylation of degron-proximal serine and threonine residues critically affects β-TrCP recognition in many substrates^29^,^30^, it would not be surprising that these residues in CREB-H-ΔTC might also have a regulatory function in β-TrCP-dependent degradation. In addition, identical or very similar β-TrCP recognizing motifs were also found in other members of the CREB3 subfamily (Fig. 2B).

### SCF^β-TrCP^ E3 ligase mediates degradation of CREB-H-ΔTC

To test whether SCF^β-TrCP^ is a bona fide E3 ubiquitin ligase responsible for CREB-H-ΔTC degradation, several lines of experiments were performed. First, we explored the interaction between CREB-H-ΔTC and SCF^β-TrCP^ by reciprocal co-immunoprecipitation. Detection of β-TrCP in the CREB-H-ΔTC immunoprecipitate and vice versa indicated the interaction between CREB-H-ΔTC and β-TrCP (Fig. 2C,D). Co-immunoprecipitation assay also indicated the association of CREB-H-ΔTC with two other components of the SCF complex: scaffold protein CUL1 and RING finger protein RBX1 (Fig. 2E,F). Simultaneous detection of β-TrCP, CUL1 and RBX1 in the CREB-H-ΔTC immunoprecipitate further suggested that CREB-H-ΔTC was bound to the SCF complex (Fig. 2G). Plausibly, β-TrCP functions as a substrate recognition protein to adapt CREB-H-ΔTC to other components of the SCF complex such as CUL1 and RBX1.

**Figure 3:**
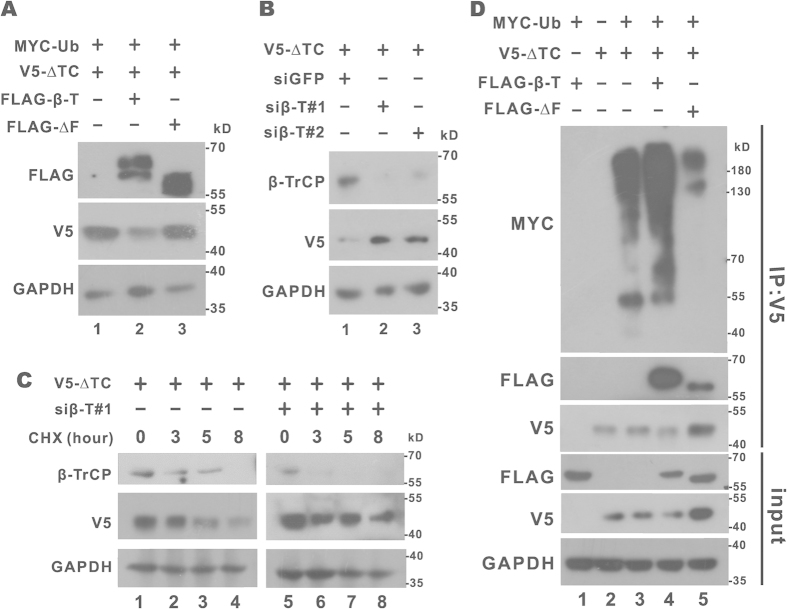
Influence of β-TrCP overexpression or suppression on steady-state level and polyubiquitination of CREB-H-ΔTC. (**A**) Immunoblotting of cell lysates derived from HEK293T cells expressing β-TrCP (FLAG-β-T) or β-TrCP-ΔFbox (FLAG-ΔF), ubiquitin (Ub) and CREB-H-ΔTC (ΔTC). (**B**) Immunoblotting of cell lysates derived from HEK293T cells transfected with two independent siRNAs targeting β-TrCP (siβ-T#1 and siβ-T#2). siGFP served as a control. (**C**) Analysis of protein stability by cycloheximide chase assay. HEK293T cells cotransfected with siβ-T#1 and CREB-H-ΔTC plasmid were treated with 200 μM cycloheximide (CHX) for 3, 5 and 8 hours before harvest. Protein samples were analyzed by SDS-PAGE and probed with the indicated antibodies. (**D**) Polyubiquitination analysis. Lysates from HEK293T cells expressing the indicated proteins were immunoprecipitated with anti-V5 and the precipitates were analyzed by immunoblotting with anti-MYC, anti-FLAG and anti-V5. The inputs (10%) were probed with anti-V5 and anti-GAPDH.

Second, we investigated how β-TrCP might influence the steady-state expression and polyubiquitination of CREB-H-ΔTC. When we expressed β-TrCP and CREB-H-ΔTC in HEK293T cells, a modest or no decrease of CREB-H-ΔTC was observed (Fig. 2C,D). Bearing in mind that ubiquitin might be limiting, we overexpressed ubiquitin in cells and found that the steady-state level of CREB-H-ΔTC was substantially reduced when β-TrCP and ubiquitin were expressed (Fig. 3A, lanes 1 and 2). In contrast, expression of β-TrCP-ΔFbox, a dominant inactive β-TrCP mutant deleted of F-box and deficient for binding with other SCF components, had no influence on the steady-state expression of CREB-H-ΔTC (Fig. 3A, lane 3). Consistent with this pattern, knockdown of β-TrCP with two independent small interfering RNAs (siRNAs) elevated the steady-state levels of CREB-H-ΔTC protein (Fig. 3B, lanes 2 and 3). Cycloheximide chase assay, in which cycloheximide was added to block *de novo* protein synthesis, was performed and the results confirmed the stabilization of CREB-H-ΔTC protein in β-TrCP knockdown cells (Fig. 3C, lanes 5–8). Furthermore, *in vivo* polyubiquitination assay indicated that the ubiquitination smear of CREB-H-ΔTC was more pronounced when β-TrCP was expressed (Fig. 3D, lanes 3 and 4). However, the expression of β-TrCP-ΔFbox decreased polyubiquitination of CREB-H-ΔTC and increased its steady-state expression (Fig. 3D, lane 5).

**Figure 4:**
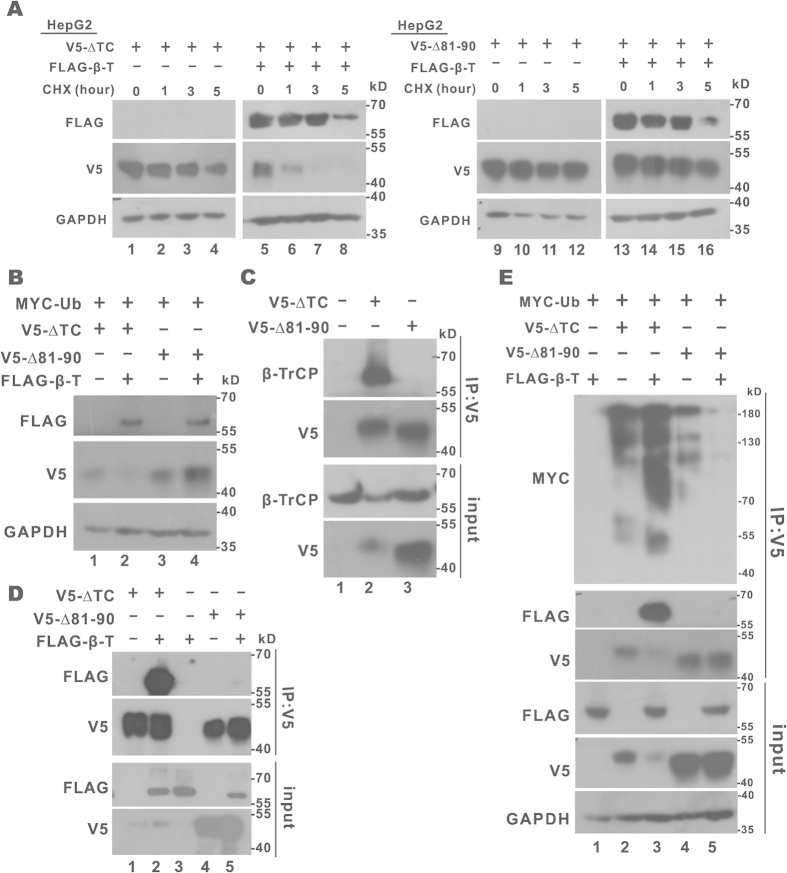
CREB-H-ΔTC-Δ81-90 was resistant to β-TrCP-induced degradation and polyubiquitination. (**A**) Protein stability. HepG2 cells expressing the indicated proteins were treated with 200 μM cycloheximide (CHX) for 1, 3 and 5 hours before harvest. Protein samples were collected, analyzed by SDS-PAGE and immunoblotted with anti-FLAG, anti-V5 and anti-GAPDH. (**B**) Steady-state protein expression. CREB-H-ΔTC (V5-ΔTC) or CREB-H-ΔTC-Δ81-90 (V5-Δ81-90), β-TrCP (FLAG-β-T) and ubiquitin (Ub) constructs were transfected into HEK293T cells. Cell lysates were immunoblotted with anti-V5 and anti-FLAG. GAPDH was detected as an internal control. (**C,D**) Immunoprecipitation. CREB-H-ΔTC (V5-ΔTC) or CREB-H-ΔTC-Δ81-90 (V5-Δ81-90) construct was transfected into HEK293T cells or HEK293T cells expressing FLAG-β-TrCP (FLAG-β-T). Cell lysates were immunoprecipitated with anti-V5. The inputs (10%) and the immunoprecipitates were analyzed by SDS-PAGE and probed with anti-V5 and either anti-β-TrCP or anti-FLAG. (**E**) *In vivo* polyubiquitination. HEK293T cells were transfected with constructs expressing the indicated proteins. Cell lysates were immunoprecipitated with anti-V5 and the precipitates were probed with anti-MYC, anti-FLAG and anti-V5. The input samples (10%) were probed with anti-FLAG, anti-V5 and anti-GAPDH.

Finally, we compared the stability of CREB-H-ΔTC in HepG2 cells with or without β-TrCP overexpression by cycloheximide chase assay. Diminution of CREB-H-ΔTC was more pronounced in the presence of β-TrCP than in its absence (Fig. 4A, lanes 6–8). Taken together, our results supported the notion that CREB-H-ΔTC is a novel substrate of SCF^β-TrCP^ E3 ubiquitin ligase.

### Characterization of β-TrCP phosphodegron in CREB-H-ΔTC

To test whether the putative β-TrCP destruction motif was indeed influential in the control of CREB-H-ΔTC stability, we deleted the DSGIS sequence and generated a CREB-H-ΔTC mutant designated CREB-H-ΔTC-Δ81–90. Three experiments were performed to characterize this mutant. First, the half-lives of CREB-H-ΔTC and CREB-H-ΔTC-Δ81–90 were compared in β-TrCP-expressing HepG2 cells. Whereas CREB-H-ΔTC was degraded rapidly in the presence of β-TrCP (Fig. 4A, lanes 6–8), the amount of CREB-H-ΔTC-Δ81–90 remained constant in β-TrCP-expressing cells treated with cycloheximide (Fig. 4A, lanes 13–16). Consistent with a longer half-life of CREB-H-ΔTC-Δ81–90 in β-TrCP-expressing cells (Fig. 4A), its steady-state level did not drop in response to β-TrCP overexpression (Fig. 4B, lanes 3 and 4). Unlike CREB-H-ΔTC-Δ81–90, CREB-H-ΔTC was almost undetectable when β-TrCP was overexpressed (Fig. 4B, lanes 1 and 2). Thus, the DSGIS motif is indeed required for β-TrCP-dependent degradation of CREB-H-ΔTC. Second, the interaction between β-TrCP and CREB-H-ΔTC-Δ81–90 was assessed by co-immunoprecipitation. The absence of endogenous or overexpressed β-TrCP in the CREB-H-ΔTC-Δ81–90 immunoprecipitate indicated the loss of β-TrCP-binding ability of the degron-deleted mutant of CREB-H-ΔTC (Fig. 4C, lane 3 and Fig. 4D, lane 5). Finally, *in vivo* polyubiquitination assay was performed and β-TrCP was found to have minimal influence on polyubiquitination of CREB-H-ΔTC-Δ81–90 (Fig. 4E, lane 5). In the control group with overexpression of β-TrCP, polyubiquitination of CREB-H-ΔTC was much more robust and CREB-H-ΔTC protein was marginally detected in both the input and the precipitate (Fig. 4E, lane 3). Hence, our results consistently demonstrated the essentiality of the DSGIS degron in β-TrCP binding, polyubiquitination and degradation of CREB-H-ΔTC.

**Figure 5:**
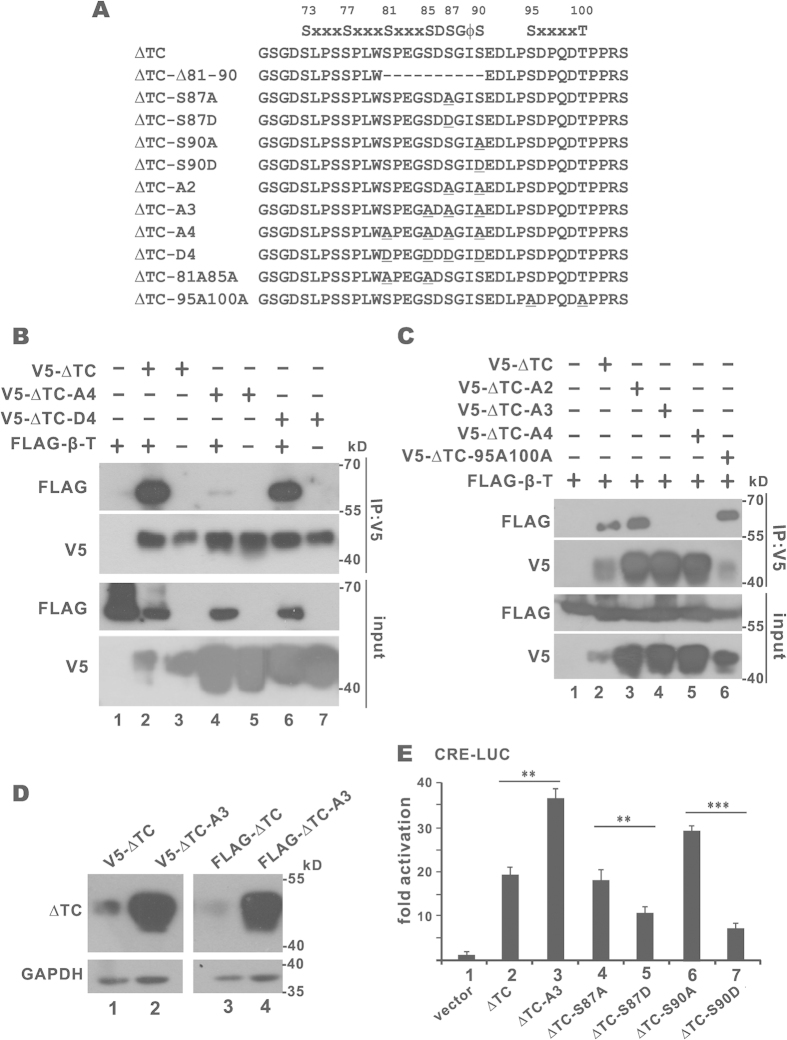
Role of phosphodegron in SCF^β-TrCP^-induced degradation of CREB-H-ΔTC. (**A**) A list of CREB-H-ΔTC mutants. The mutation sites are underlined. (**B,C**) Co-immunoprecipitation. Plasmids expressing the indicated proteins were transfected into HEK293T cells. Cell lysates were immunoprecipitated with anti-V5. Both inputs (10%) and precipitates were probed with anti-FLAG and anti-V5. (**D**) Immunoblotting. (**E**) Luciferase reporter assay. Expression plasmids for CREB-H-ΔTC (ΔTC) and its mutants were individually transfected with luciferase reporter plasmid pCRE-Luc into HEK293T cells. The values of firefly luciferase were measured by luminescence and normalized with readings for *Renilla* luciferase. Data represent the means ± standard deviations of three independent measurements. Two tailed Student’s t test was performed to evaluate the statistical significance of the differences between the indicated groups. *p < 0.05. **p < 0.01. ***p < 0.001.

For most β-TrCP substrates, phosphorylation of the β-TrCP degron is usually required for their binding to β-TrCP^29^,^30^. With this in mind we constructed and characterized different phosphorylation mutants of CREB-H-ΔTC (Fig. 5A) to determine whether the interaction between β-TrCP and CREB-H-ΔTC is phosphorylation-dependent.

First, we generated a non-phosphorylatable mutant CREB-H-ΔTC-A4, in which four serine residues S81, S85, S87 and S90 within or near the DSGIS degron had been replaced by alanine. On the contrary, a phosphomimetic mutant CREB-H-ΔTC-D4 with the same four serine residues substituted by aspartate was also made (Fig. 5A). We then compared the β-TrCP-binding ability of CREB-H-ΔTC, CREB-H-ΔTC-A4 and CREB-H-ΔTC-D4. In sharp contrast to CREB-H-ΔTC-A4, which lost its β-TrCP-binding activity almost completely (Fig. 5B, lane 4), CREB-H-ΔTC-D4 was fully competent for binding with β-TrCP (Fig. 5B, lane 6). These results supported the requirement of degron phosphorylation for β-TrCP recognition.

Second, further mutational analysis was carried out to identify the serine residues, the phosphorylation of which would be critical for β-TrCP recognition. Interestingly, whereas CREB-H-ΔTC-A3 with non-phosphorylatable S85A, S87A and S90A did not interact with β-TrCP, CREB-H-ΔTC-A2 carrying non-phosphorylatable S87A and S90A was still capable of binding to β-TrCP (Fig. 5C, lanes 3 and 4). Considered together the full ability of CREB-H-ΔTC-S81A S85A harboring non-phosphorylatable S81A and S85A to bind with β-TrCP (Supplementary Figure 1, lane 7), these results were compatible with one model in which phosphorylation at S85, S87 and S90 might be minimally required for β-TrCP binding. In other words, the β-TrCP-recognizing motif in CREB-H-ΔTC is SDSGIS. Consistent with its loss of β-TrCP-binding ability, the steady-state level (Fig. 5D, lanes 2 and 4) and transcriptional activity (Fig. 5E, group 3) of CREB-H-ΔTC-A3 were elevated. Additionally, although less S95A T100A mutant was detected in the precipitate, the mutation had minimal impact on the binding with β-TrCP (Fig. 5C, lane 6). Thus, these sites might not be critical in the regulation of β-TrCP recognition.

Finally, we explored how phosphorylation within the SDSGIS degron might affect transcriptional activity of CREB-H-ΔTC. S87 and S90 within the SDSGIS motif were replaced one by one by alanine and aspartate (Fig. 5A). Luciferase reporter assay was performed on the CRE promoter which is known to be highly responsive to CREB-H-ΔTC. CREB-H-ΔTC-S90A displayed increased transcriptional activity but the CREB-H-ΔTC-S90D mutation had inhibitory effect (Fig. 5E, groups 6 and 7). CREB-H-ΔTC-S87A was also transcriptionally more active than CREB-H-ΔTC-S87D (Fig. 5E, groups 4 and 5). The activity difference between non-phosphorylatable and phosphomimetic mutants of CREB-H-ΔTC suggested that phosphorylation at S87 and S90 might indeed be critical in governing β-TrCP recognition.

### S73 and S77 are degron-proximal inhibitory phosphorylation sites of CREB-H-ΔTC

The presence of evolutionarily conserved serine residues in the flanking regions of the β-TrCP phosphodegron in CREB-H-ΔTC (Fig. 2A) prompted us to ask whether their phosphorylation might have regulatory roles in β-TrCP-dependent ubiquitination and degradation of CREB-H-ΔTC. In this regard, we noted that phosphoserines and phosphothreonines adjacent to the degron in other β-TrCP substrates such as Snail, YAP and Nrf2 are also known to be influential in substrate recognition and degradation^39^,^40^,^41^.

**Figure 6:**
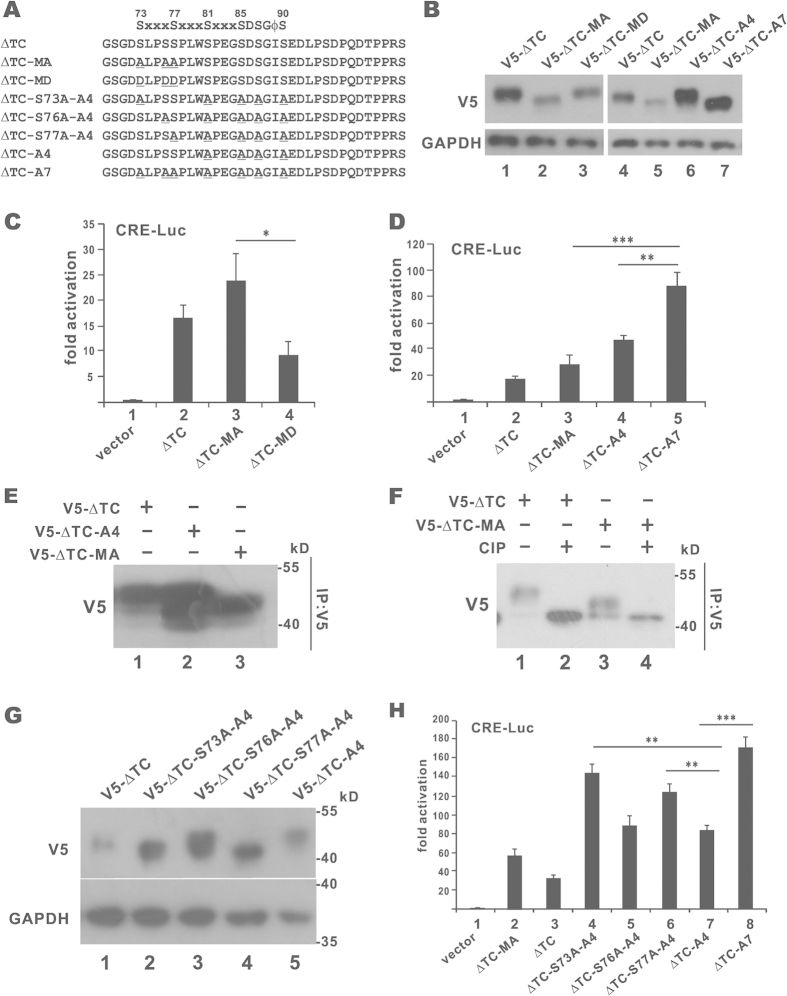
Influence of phosphodegron-proximal serine residues on CREB-H-ΔTC degradation. (**A**) A list of CREB-H-ΔTC mutants. The mutation sites were underlined. (**B**) Protein expression. Immunoblotting of cell lysates derived from HEK293T cells transfected with CREB-H-ΔTC and mutants was performed with anti-V5 and anti-GAPDH. (**C,D**) Luciferase reporter assay. Luciferase reporter assay was carried out as in Fig. 5E. (**E**) Immunoprecipitation. HEK293T cells expressing CREB-H-ΔTC (V5-ΔTC) or its mutants were treated with 10 μM MG132 for 3 hours before harvest. Protein samples were immunoprecipitated and probed with anti-V5. (**F**) CIP treatment. V5-CREB-H-ΔTC and V5-CREB-H-ΔTC-MA were transiently expressed in HEK293T cells. The protein lysates were immunoprecipitated with anti-V5. The precipitates were mock treated or treated with calf intestinal alkaline phosphatase (CIP) for 1 hour at 37 °C and analyzed by immunoblotting with anti-V5. (**G**) Immunoblotting of different CREB-H-ΔTC mutants. (**H**) Luciferase reporter assay. Luciferase reporter assay was performed as in Fig. 5E.

To shed light on the roles of serine phosphorylation in the region of amino acid residues 73–77 proximal to the degron, we constructed non-phosphorylatable mutant CREB-H-ΔTC-MA, in which S73, S76 and S77 had been replaced by alanine. Opposite to this, a CREB-H-ΔTC-MD mutant with serine-to-aspartate substitution at the same three positions was also created (Fig. 6A). Notably, CREB-H-ΔTC-MA migrated faster on SDS-PAGE than CREB-H-ΔTC, whereas no noticeable change in the electrophoretic mobility rate of CREB-H-ΔTC-MD was found (Fig. 6B, lanes 2 and 3). The electrophoretic mobility shift indicated that one or more sites among S73, S76 and S77 might be post-translationally modified. Constitutive phosphorylation was most probable since the phosphomimetic form migrated as fast as the wild-type protein. Luciferase reporter assay confirmed the rise and fall of the transcriptional activity of CREB-H-ΔTC-MA and CREB-H-ΔTC-MD, respectively (Fig. 6C, groups 3 and 4), indicating that phosphorylation of this region affects protein function.

We generated another mutant named CREB-H-ΔTC-A7, which contains both A4 and MA mutations (Fig. 6A). This mutant was not only as stable as CREB-H-ΔTC-A4 but also migrated as fast as CREB-H-ΔTC-MA (Fig. 6B, lanes 5–7). In addition, CREB-H-ΔTC-A7 had much higher transcriptional activity on the CRE-Luc reporter than CREB-H-ΔTC-A4 (Fig. 6D, groups 4 and 5), indicating the enhancing effect of the MA mutation on CREB-H-ΔTC-dependent transcription. When we used anti-V5 to pull down CREB-H-ΔTC and its mutants from cells, multiple protein species were found in the precipitates (Fig. 6E). Treatment with calf intestine phosphatase eliminated the multiple protein bands in the CREB-H-ΔTC and CREB-H-ΔTC-MA immunoprecipitates (Fig. 6F, lanes 2 and 4), providing crucial support to the phosphorylation of CREB-H-ΔTC.

To identify the exact phosphorylation sites among S73, S76 and S77, additional mutants were generated and tested. Because single mutants CREB-H-ΔTC-S73A, CREB-H-ΔTC-S76A and CREB-H-ΔTC-S77A only had weak phenotypes (data not shown), we introduced them in the background of CREB-H-ΔTC-A4 (Fig. 6A). Fast-migrating species of both CREB-H-ΔTC-S73A-A4 and CREB-H-ΔTC-S77A-A4 were observed in SDS-PAGE (Fig. 6G, lanes 2 and 4). In contrast, fast migration was not observed for CREB-H-ΔTC-S76A-A4, CREB-H-ΔTC-A4 or CREB-H-ΔTC (Fig. 6G, lanes 1, 3 and 5). Consistent with the electrophoretic mobility patterns, transcriptional activities of CREB-H-ΔTC-S73A-A4 and CREB-H-ΔTC-S77A-A4 on the CRE-Luc reporter were higher than that of CREB-H-ΔTC-A4 and close to that of CREB-H-ΔTC-A7 (Fig. 6H, groups 4, 6, 7 and 8). Taken together, our data suggested that both S73 and S77 are inhibitory phosphorylation sites of CREB-H-ΔTC.

### Stabilized CREB-H-ΔTC exhibits enhanced transcriptional activity on target genes

**Figure 7:**
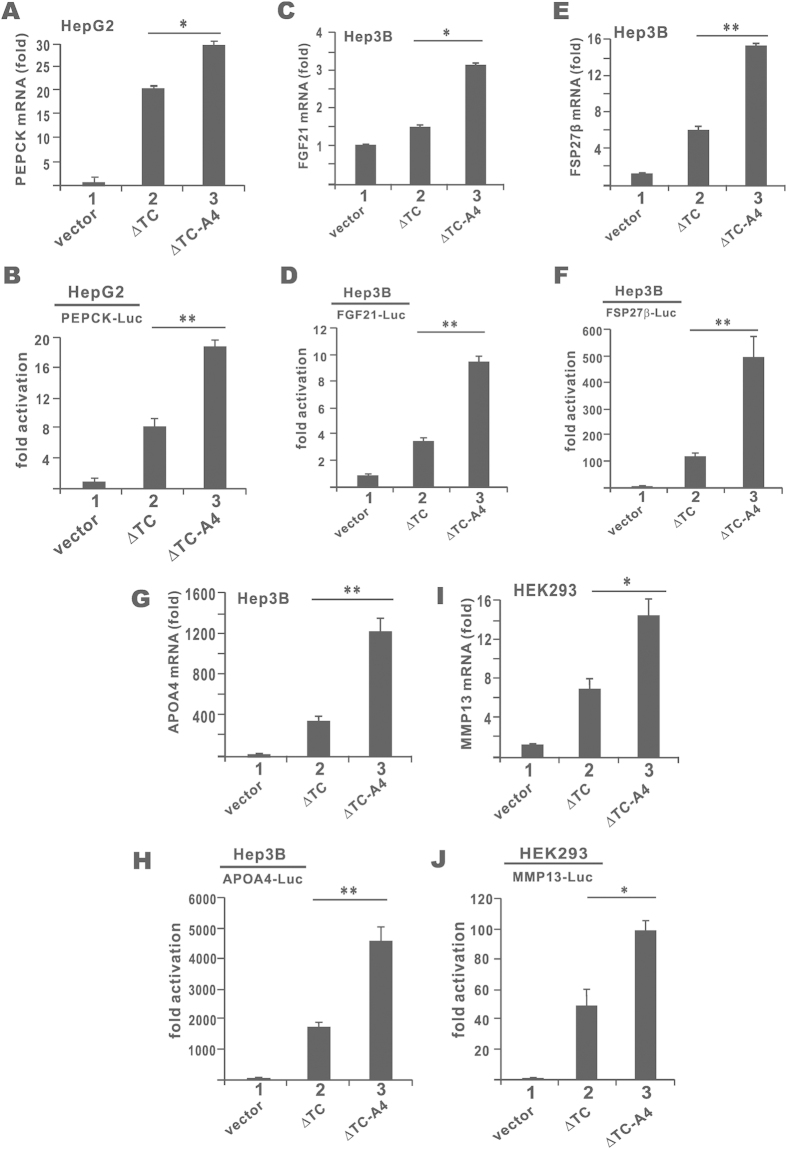
Transcriptional activity of CREB-H-ΔTC and its mutant on target genes. PEPCK, FGF21, FSP27β, APOA4 and MMP13 mRNA expression in transfected HepG2, Hep3B or HEK293 cells were analyzed by RT-qPCR (panels **A,C,E,G,I**). Relative mRNA expression levels were normalized to the levels of β-tubulin transcript. Fold activation values represent the means ± standard deviations of three independent measurements. Concurrently, luciferase reporter assays were performed with the indicated reporters in HepG2, Hep3B and HEK293 cells as in Fig. 5E (panels **B,D,F,H,J**). Results are representative of triplicate experiments and error bars indicate the standard deviations. Statistical analysis was performed with two tailed Student’s t test. *p < 0.05. **p < 0.01.***p < 0.001.

Above we demonstrated a stabilizing effect of the A4 mutation on CREB-H-ΔTC (Fig. 6B, lane 6). This also correlated with a potentiating effect of the A4 mutation on the transcriptional activity of CREB-H-ΔTC on the CRE-Luc reporter (Fig. 6D, group 4). In this part of our study we made use of the CREB-H-ΔTC-A4 mutant to investigate the impact of CREB-H-ΔTC stabilization on target gene transcription in cultured cells. We and others have previously identified phosphoenolpyruvate carboxykinase (PEPCK) gene as a target of CREB-H in the regulation of gluconeogenesis^4^,^7^. In addition, three genes critically involved in lipid metabolism, namely FGF21, FSP27β and APOA4, have recently been found to be CREB-H target genes in the liver^12^,^18^,^20^. On the other hand, CREB-H-ΔTC has also been shown to induce cell secretion and activate MMP13 gene transcription in HEK293 cells^14^. Hence, we chose these five genes for analysis of the transcriptional activity of CREB-H-ΔTC-A4. CREB-H-ΔTC was capable of activating the transcription of PEPCK, FGF21, FSP27β and APOA4 in HepG2 or Hep3B hepatoma cells as well as that of MMP13 in HEK293 cells, but the stimulating effect of CREB-H-ΔTC-A4 mutant was more pronounced on all five genes (Fig. 7A,C,E,G,I, groups 2 and 3). Consistent with the RT-qPCR results, the transcriptional activity of CREB-H-ΔTC-A4 as reflected in the expression of luciferase reporter driven by all five promoters was also higher than that of CREB-H-ΔTC (Fig. 7B,D,F,H,J, groups 2 and 3). Plausibly, stabilization of CREB-H-ΔTC led to enhancement of transcriptional activation of target genes both in magnitude and in duration.

**Figure 8:**
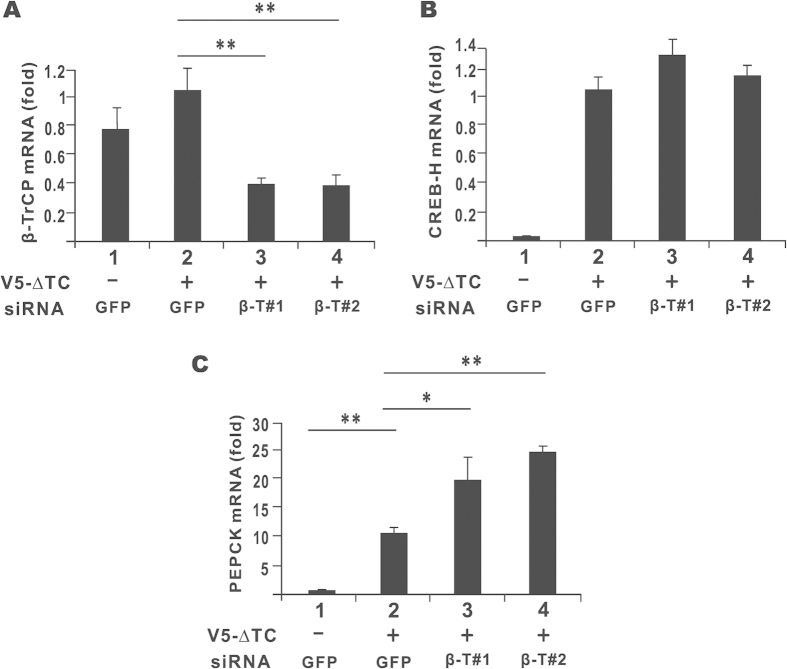
PEPCK gene expression in β-TrCP-depleted cells. The expression of PEPCK, β-TrCP and CREB-H transcripts in HepG2 cells transfected with two independent siRNAs targeting β-TrCP (siβ-T#1 and siβ-T#2) and CREB-H-ΔTC (V5-ΔTC) construct. Relative mRNA expression levels were normalized to the levels of β-tubulin transcript. Fold activation values represent the means ± standard deviations of three independent experiments. Statistical analysis was performed with two tailed Student’s t test. *p < 0.05. **p < 0.01.***p < 0.001.

To further verify the role of β-TrCP in the regulation of PEPCK gene expression, the two β-TrCP-targeting siRNAs were used to deplete endogenous β-TrCP expression in HepG2 cells. The knockdown effect was highly specific to β-TrCP and the siRNAs had no influence on the expression of CREB-H mRNA (Fig. 8A,B). By contrast, the expression of PEPCK transcript induced by CREB-H-ΔTC was significantly boosted when endogenous β-TrCP was depleted (Fig. 8C, groups 3 and 4). Thus, the activation of PEPCK gene expression by CREB-H was regulated by β-TrCP.

## Discussion

**Figure 9:**
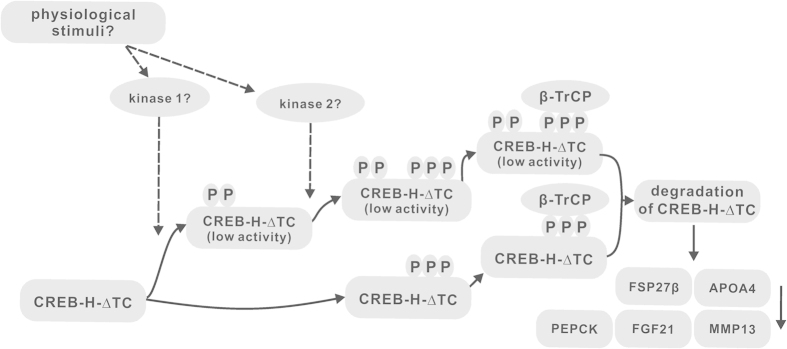
Working model for negative regulation of CREB-H-dependent transcription. Two kinases such as GSK-3 and CKII might be activated in response to physiological stimuli. The β-TrCP recognizing motif and its N-terminal adjacent region within CREB-H-ΔTC are phosphorylated. Phosphorylated CREB-H-ΔTC has low activity and is ultimately degraded through a β-TrCP-dependent pathway, leading to down regulation of the expression of CREB-H target genes.

In the present study, we uncovered a new mechanism by which SCF^β-TrCP^ ubiquitin ligase mediates the ubiquitination and degradation of CREB-H-ΔTC, the active form of liver-enriched transcription factor CREB-H. An evolutionarily conserved β-TrCP phosphodegron with the SDSGIS motif was identified and characterized. In addition, the degron-proximal S73 and S77 were identified as the inhibitory phosphorylation sites for β-TrCP-dependent ubiquitination and degradation. Based on our findings, we propose a model for regulation of CREB-H-ΔTC activity (Fig. 9). In response to as yet uncharacterized physiological stimuli, CREB-H-ΔTC is phosphorylated sequentially by two kinases, the second of which might be casein kinase II (CKII) as demonstrated in another study^38^. Phosphorylation by CKII at the degron is required for β-TrCP binding and recognition. According to our results, phosphorylation at three serine residues S85, S87 and S90 might be minimally required. In addition, inhibitory phosphorylation at S73 and S77 within the degron-proximal region, plausibly by glycogen synthase kinase 3 (GSK-3) distinct from CKII as suggested in the other study^38^, also has regulatory function. CREB-H-ΔTC phosphorylated at S73 and S77 has low activity and is probably sensitized to subsequent degron phosphorylation by CKII leading to K48-linked polyubiquitination and proteasome-mediated degradation. As a result, transcription of CREB-H target genes, such as PEPCK, FGF21, FSP27β, APOA4 and MMP13, is down-regulated.

At the completion of our study, another work documenting SCF^β-TrCP^-dependent degradation of CREB-H-ΔTC was published^38^. The two studies came to the same conclusion. The results presented were largely consistent and complementary. However, most of the mutants constructed and analyzed, some of the results obtained, as well as the emphases of the two papers were different. Whereas two kinases CKII and GSK-3 were identified in the other study, K48-linked polyubiquitination of CREB-H-ΔTC was analyzed in more detail in our work. On the other hand, the S87A S90A mutant was largely deficient for binding with β-TrCP in the other study^38^. In contrast, the β-TrCP-binding activity was unaffected in the same CREB-H-ΔTC-A2 mutant in our work, but a triple mutant CREB-H-ΔTC-A3 (i.e. S85A, S87A and S90A) lost the capability to bind with β-TrCP (Fig. 5C). Our results therefore suggested that S85, S87 and S90 in CREB-H-ΔTC might be minimally required for β-TrCP recognition. We are currently performing animal studies to clarify whether the CREB-H-ΔTC-A3 and CREB-H-ΔTC-A4 mutants are constitutively active and β-TrCP-dependent CREB-H-ΔTC degradation is physiologically relevant *in vivo*.

Although the two studies corroborated with each other to support the new model for SCF^β-TrCP^-dependent ubiquitination and degradation of CREB-H-ΔTC, several questions remain unanswered. First, direct evidence for S85 phosphorylation is required although we have demonstrated the essential role of this residue in β-TrCP binding. Additional phosphorylation sites including S81, S95 and T100 also merit further analysis. Second, in addition to CKII and GSK-3 reported in the other study^38^, other kinases acting on the SDSGIS motif and its flanking regions remain to be identified and characterized. Based on bioinformatic analysis, we speculate that S73 and S77 might be phosphorylated by proline-dependent kinases in addition to GSK-3, whereas S87 and S90 might be targeted by acidophilic serine kinases in addition to CKII^42^. Finally, the mechanism by which dephosphorylation of S73 and S77 augments CREB-H-ΔTC activity remains unclear. In this regard, we have compared the subcellular localization of CREB-H-ΔTC and CREB-H-ΔTC-MA but both were found in the nucleus (data not shown), excluding the possibility of cytoplasmic sequestration of CREB-H-ΔTC-MA. Other mechanisms should be explored in future study.

The relationship between the β-TrCP phosphodegron and its proximal region warrants further investigations. One possibility is that phosphorylation of the degron-proximal region “primes” or sensitizes degron phosphorylation. Such “priming phosphorylation” has been found in many other β-TrCP substrates^39^,^40^,^43^. One example is Snail with a **DS**_**(0)**_**GxxS**_**(+4)**_xxxS_(+8)_xxS_(+11)_ motif, in which the β-TrCP degron is bolded. In this case, priming phosphorylation at the serine residue at +11 position is required for subsequent phosphorylation at serine residues at +8, +4 and 0 positions^39^,^44^. Another example is YAP, which contains an S_(−3)_T**DS**_**(0)**_**GϕS**_**(+3)**_ sequence. Here S_(−3)_ served as a priming phosphorylation site for S_(0)_ and S_(+3)_^40^. Considering that CREB-H-ΔTC contains an S_(−14)_xxxS_(−10)_xxxS_(−6)_xxxS_(−2)_**DS**_**(0)**_**GϕS**_**(+3)**_ sequence, S_(−14)_xxxS_(−10)_xxxS_(−6)_ plausibly functions as the priming phosphorylation signal for subsequent S_(−2)_DS_(0)_GϕS_(+3)_ phosphorylation. Another possibility is that CREB-H-ΔTC, like CREB, contains a kinase-inducible domain^45^. Multiple kinases might phosphorylate S_(−14)_xxxS_(−10)_xxxS_(−6)_xxxS_(−2)_**DS**_**(0)**_**GϕS**_**(+3)**_xxxxS_(+8)_xxxxT_(+13)_ region to regulate CREB-H-ΔTC activity and stability.

The phosphodegron of CREB-H-ΔTC, DS_(0)_GϕS_(+3)_, is very similar to but does not exactly match the canonical β-TrCP-binding motif DS_(0)_GϕxS_(+4)_. However, several substrates without the x residue, such as RV-NSP1, YAP and Nrf2, have been identified, indicating that the x residue is not essential^40^,^41^,^46^. Interestingly, residue at −2 position of DS_(0)_GϕS_(+3)_ always prefers phosphorylatable residues (S, T or Y). Experimentally, we also demonstrated the requirement of S85 for the binding of CREB-H-ΔTC to β-TrCP. Thus, we propose the phosphorylatable residue at −2 position should be important for the binding of the SDSGϕS motif to β-TrCP. In other words, phosphorylation at −2 position might be “priming” degron phosphorylation at other sites. Alternatively, phosphorylation at −2 position could provide a negative charge to increase the binding of the SDSGϕS motif to β-TrCP. Previous studies have shown that negatively charged residue is most critical to β-TrCP binding^47^.

We have found that CREB3L4 contains the same β-TrCP recognizing sequence (SDSGIS) as CREB-H. In addition, the SDSDGS motif in CREB3L1 and the SDSEGS motif in CREB3L2 are very similar to the motif found in CREB-H. Plausibly, β-TrCP-mediated degradation is a common mechanism shared by most of these proteins. CREB3L1 and CREB3L2 are the substrates of Fbxw7. Both Fbxw7 and β-TrCP are F-box proteins of the SCF E3 ubiquitin ligase and they share many similar properties. Fbxw7 recognizes an S/TxxxS/T motif, in which S/T are phosphorylated^48^. It will be of interest to see whether Fbxw7 might also target CREB-H and CREB3L4. Interestingly, within CREB3L2 and CREB3L1 sequence, the reported Fbxw7-binding motif (TPPSS) is immediately next to the putative β-TrCP-binding motif (Fig. 2B). Fbxw7 and β-TrCP can cooperatively degrade substrates^49^. Thus, it will be intriguing to determine whether Fbxw7 and β-TrCP might cooperatively mediate the degradation of some CREB3 subfamily proteins.

The expression and activity of CREB-H are regulated at multiple levels including proteolytic activation, phosphorylation, ubiquitination and degradation. Most of the results are obtained with CREB-H-ΔTC in our study and in the other work^38^. CREB-H-ΔTC is the physiologically active form of CREB-H. The full-length CREB-H tethered to the ER membrane is an inactive protein with no transcriptional activity. However, since the phosphodegron and flanking regions are present in both CREB-H and CREB-H-ΔTC, it is not surprising if CREB-H degradation is regulated through the same mechanism. Indeed, we showed that CREB-H was also subjected to K48-linked polyubiquitination. Thus, CREB-H might also be targeted by β-TrCP in addition to other ER-bound E3 enzymes involved in ERAD. β-TrCP is an E3 ligase that plays an important role in the regulation of various cell signaling pathways governing cell division, metabolism and oncogenesis^29^,^31^. Thus, our demonstration of β-TrCP-dependent ubiquitination and degradation of CREB-H-ΔTC provides new opportunities to explore how CREB-H degradation and activity are linked to other signal transduction pathways converging on β-TrCP. β-TrCP has been suggested as an important new target for anti-cancer therapy^27^. By the same reasoning, our findings also reveal a new strategy of controlling CREB-H activity by enhancing or decreasing the expression and activity of β-TrCP. For example, small-molecule activators and inhibitors of the two kinases that phosphorylate CREB-H-ΔTC could be harnessed for therapeutic modulation of the expression of CREB-H target genes.

## Methods

### Plasmids and primary antibodies

Expression plasmids pcDNA3.1-V5-CREB-H and pcDNA3.1-V5-CREB-H-ΔTC for human CREB-H and CREB-H-ΔTC have been previously described^4^,^11^. Plasmid pCMV-Tag2B-CREB-H-ΔTC was constructed by subcloning human CREB-H-ΔTC cDNA into pCMV-Tag2B (Agilent). Plasmid pcDNA3-FLAG-β-TrCP was kindly provided by Dr. Peter Howley (Harvard Medical School, MA, USA)^50^. β-TrCP cDNA was subcloned into pCAGEN vector with a V5 tag. pCMV-MYC-Ub and its mutant forms were derived from gifts of plasmids from Dr. Dirk Bohmann (University of Rochester Medical Center, NY, USA), Dr. Ted Dawson (Johns Hopkins University School of Medicine, MD, USA) and Dr. James Chen (University of Texas Southwestern Medical Center, TX, USA)^51^,^52^,^53^. pcDNA3-MYC3-CUL1 and pcDNA3-MYC3-RBX1 were supplied by Dr. Yue Xiong (School of Medicine, University of North Carolina at Chapel Hill, NC, USA)^54^,^55^. The promoter regions of FGF21 (−1033 ~ + 150), FSP27β (−808 ~ + 65), APOA4 (−763 ~ + 104) and MMP13 (−1659 ~ + 23) were amplified from human genomic DNA and cloned into pGL3-basic (Promega). Primer sequences used for cloning are listed in Supplementary Table 1. Plasmid pSV-RLuc was purchased from Promega. Plasmids pCRE-Luc and pPEPCK-Luc have been described previously^4^.

Plasmid FLAG-β-TrCP-ΔFbox and plasmids expressing various CREB-H-ΔTC mutants were generated by site-directed mutagenesis using reagents supplied by Agilent. Mutagenic primers were designed using the web-based QuikChange Primer Design Program (www.agilent.com/genomics/qcpd; Agilent).

Primary antibodies used in this study included mouse anti-V5 (Invitrogen), rabbit anti-FLAG (F7425; Sigma-Aldrich), mouse anti-FLAG (M2; Sigma-Aldrich), rabbit anti-MYC (A-14; Santa Cruz), rabbit anti-glyceraldehyde-3-phosphatase dehydrogenase (anti-GAPDH: H12; Santa Cruz), rabbit anti-β-TrCP (D13F10; Cell Signaling) and rabbit anti-β-actin (Sigma-Aldrich).

### Cell culture and transfection

Human hepatoma cell line Hep3B and human embryonic kidney cell lines HEK293 and HEK293T were cultured in Dulbecco’s Modified Eagle’s Medium containing 10% fetal bovine serum (Life Technologies). Human hepatoma cell line HepG2 was grown in Eagle’s Minimum Essential Medium (Life Technologies) supplemented with 10% fetal bovine serum. Transfection of cells was performed as previously described^56^,^57^.

For proteasome inhibition, transfected cells were treated with 10 μM MG132 (Calbiochem) for 6 hours before harvest. For protein turnover assay, 200 μM cycloheximide (Sigma-Aldrich) was added into transfected cells 5 hours before harvest.

### Immunoblotting

Forty-eight hours after transfection, cells were harvested in NP40 buffer (50 mM Tris-Cl, pH7.4, 250 mM NaCl, 1 mM EDTA, 1% NP-40 and 0.2% Triton X-100) supplemented with protease inhibitor cocktails (Roche). Protein concentration of cell lysates was determined by Bradford method (BioRad). Protein samples were separated by SDS-PAGE, electroblotted onto polyvinylidene difluoride membranes (Millipore), incubated with primary and secondary antibodies, and visualized by enhanced chemiluminescence (Amersham).

### Co-immunoprecipitation, phosphatase treatment and *in vivo* polyubiquitination

Co-immunoprecipitation was carried out as described^58^. Cells were lysed with NP40 buffer supplemented with protease inhibitor cocktails. Antibodies were recovered by incubating with recombinant protein G agarose (Invitrogen) for 2 hours, followed by overnight incubation with the remaining cell lysate at 4 °C. The protein G agarose was collected and washed at least three times with wash buffer (50 mM Tris-Cl, pH7.4, 800 mM NaCl, 1 mM EDTA, 1% NP-40 and 0.2% Triton X-100) after incubation. The immunoprecipitates were separated by SDS-PAGE and analyzed by immunoblotting.

Phosphatase assay was performed as described^59^. HEK293T cells were cultured in 60 mm dishes and transiently transfected with V5-CREB-H-ΔTC or its mutant forms. The protein lysates were incubated with mouse anti-V5 antibody and recombinant protein G agarose overnight. Next day the beads were rinsed with cold PBS and treated 10 units of calf intestine alkaline phosphatase (Fermentas) for 1 hour at 37 °C. After treatment, the beads were pelleted, washed twice with wash buffer and analyzed by immunoblotting.

For *in vivo* polyubiquitination, cells were co-transfected with the MYC-ubiquitin plasmid in combination with the wide-type or mutant V5-CREB-H-ΔTC plasmids. Forty-eight hours after transfection, total protein was collected, immunoprecipitated with anti-V5 antibody, resolved with SDS-PAGE and immunoblotted with anti-MYC antibody.

### Luciferase reporter assay

Cells were harvested 36 hours after transfection for dual luciferase assay as previously described^4^,^11^. Transcriptional activity on cAMP response elements (CRE) as well as PEPCK, FGF21, FSP27β, APOA4 and MMP13 promoters was measured with reporter plasmids pCRE-Luc, pPEPCK-Luc, pFGF21-Luc, pFSP27β-Luc, pAPOA4-Luc and pMMP13-Luc, respectively. Transfection efficiencies were normalized using a control plasmid pSV-RLuc.

### RNA interference

RNA knockdown experiments were performed as described^57^. HepG2 and HEK293T cells were transfected with 100 nM siRNA using Lipofectamine 2000 (Invitrogen). The siRNA sequences are as follows: 5′-AAGUGGAAUU UGUGGAACAU CdTdT-3′ for siβ-TrCP#1^43^; 5′-GAGAGAGAAG ACUGUAAUAdT dT-3′ for siβ-TrCP#2 and 5′-GCUACCUGUU CCAUGGCCAdT dT-3′ for siGFP^60^,^61^.

### Real-time PCR analysis

Total RNA was extracted from cells using the RNAiso Plus reagent (Takara) according to the manufacturer’s instructions and then incubated with DNase I (Ambion) to remove remaining genomic DNA. Reverse transcription was performed using the Transcriptor First Strand cDNA Synthesis reagents (Roche). Real-time quantitative PCR was conducted in the presence of SYBR Premix Ex Taq (Takara) with the StepOnePlus real-time PCR system (Applied Biosystems). The mRNA level of β-tubulin was determined as an internal control and the results were analyzed using StepOne Software v2.3 (Applied Biosystems). Primer sequences used are listed in Supplementary Table 1.

## Additional Information

**How to cite this article**: Cheng, Y. *et al.* β-TrCP-mediated ubiquitination and degradation of liver-enriched transcription factor CREB-H. *Sci. Rep.* **6**, 23938; doi: 10.1038/srep23938 (2016).

## Supplementary Material

Supplementary Information
