# Supplementary material for: β-TrCP-mediated ubiquitination and degradation of liver-enriched transcription factor CREB-H
Source: Sci Rep. 2016 Mar 31;6:23938. doi: 10.1038/srep23938 (PMC4814919; doi:10.1038/srep23938)
Supplement: Supplementary Information [file srep23938-s1.pdf]

Supplementary information

β-TrCP-Mediated Ubiquitination and Degradation of Liver-Enriched Transcription Factor CREB-H

Yun Cheng, Wei-Wei Gao, Hei-Man Vincent Tang, Jian-Jun Deng, Chi-Ming Wong, Chi-Ping Chan & Dong-Yan Jin

Supplementary Table 1. List of primers used in the study

Primer name	Forward	Reverse
Primers used for promoter cloning		
FGF21 promoter	GGGGTACCCACCCACGAGTCCAGATCCCT	CCCAAGCTTCAATGGCTCGGGTCCTCAGGT
APOA4 promoter	GGGGTACCGAGATGACCGTACTACCCACT	CCCAAGCTTCCTGAGCTGCTTGCTGGGCTG
FSP27β promoter	GGGGTACCGGAACTATCTGTGCAAAGGGTTG	CCCAAGCTTCCCCTGGCTCTGGTCACA
MMP13 promoter	GGGGTACCAAGCTTAGTCGATGAATCAAACT	CCGCTCGAGCTTGAATGGTGATGCCTGGG
Primers used for real-time RT-qPCR		
PEPCK	CCCAGGCAGTGAGGGAGTTT	AGTGAGAGCCAACCAGCAGT
FGF21	GACCAGAGCCCCGAAAGTC	TGTATCCGTCCTCAAGAAGCAG
APOA4	ACGGTGATGTGGGACTACTT	AGAGGGCATTGAGTTGCTGG
MMP13	TCCTGAAGAGCATTTGGGGTAA	GGTTCCAGCCACGCATAGTC
FSP27β	CCAGAGCCAGGGGATGAGAA	TGGAGAGGGACTTGGGGTAG
β-tubulin	GGACGAGATGGAGTTCACCG	GAGGAAAGGGGCAGTTGAGT

All primer sequences are listed from 5′ to 3′. Restriction sites are underlined. FSP27β primers have been described previously[20].

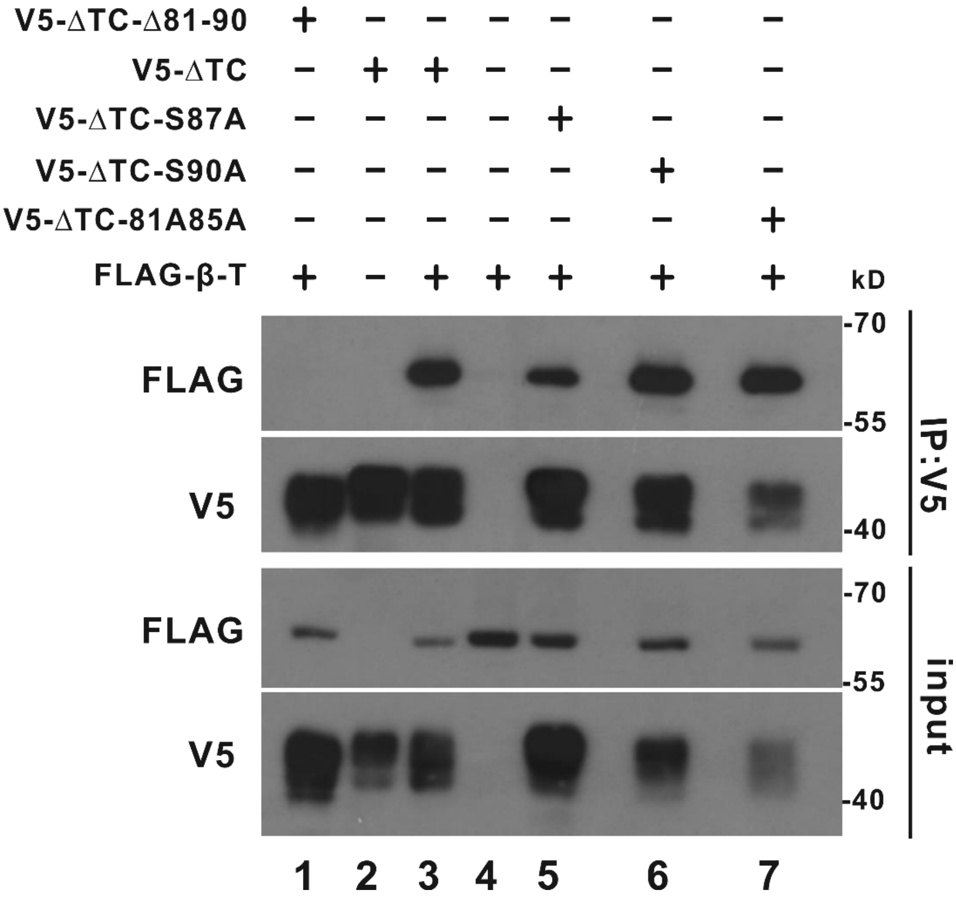

Supplementary Figure 1. Mapping of serine residues in the phosphodegron required for SCF$^{\beta\text{-TrCP}}$-induced degradation of CREB-H-ΔTC. Plasmids expressing the indicated proteins were transfected into HEK293T cells. Cell lysates were immunoprecipitated with anti-V5. Both inputs (10%) and precipitates were analyzed by Western blotting with anti-FLAG and anti-V5.
